# Construction and internal validation of a predictive model for risk of gastrointestinal bleeding in children with abdominal Henoch-Schönlein purpura: A single-center retrospective case-control study

**DOI:** 10.3389/fimmu.2022.1025335

**Published:** 2022-09-29

**Authors:** Lingli Sun, Wenjuan Liu, Changjian Li, Yong Zhang, Yuanyuan Shi

**Affiliations:** ^1^ Department of Child Health, Wuhan Children’s Hospital (Wuhan Maternal and Child Healthcare Hospital), Tongji Medical College, Huazhong University of Science and Technology, Wuhan, China; ^2^ Department of Rheumatology and Immunology, Wuhan Children’s Hospital (Wuhan Maternal and Child Healthcare Hospital), Tongji Medical College, Huazhong University of Science and Technology, Wuhan, China; ^3^ Department of Cardiology, Wuhan Children’s Hospital (Wuhan Maternal and Child Healthcare Hospital), Tongji Medical College, Huazhong University of Science and Technology, Wuhan, China; ^4^ Department of General Medicine, Wuhan Fourth Hospital, Puai Hospital, Wuhan, China

**Keywords:** Henoch-Schönlein purpura, IgA vasculitis, gastrointestinal involvement, gastrointestinal bleeding, predictive scoring model, binary logistic regression

## Abstract

Early identification of gastrointestinal (GI) bleeding in children with abdominal Henoch-Schönlein purpura (HSP) is essential for their subsequent treatment, and a risk prediction model for GI bleeding in abdominal HSP was constructed in this study to assist physicians in their decision-making. In a single-center retrospective study, the children collected were divided into two parts, a training set and a validation set, according to the time of admission. In the training set, univariate analysis was performed to compare demographic data and laboratory tests between the two groups of children with GI and non-GI bleeding, and the independent risk factors were derived using binary logistic equations to develop a scoring model for predicting GI bleeding in children by odds ratio (OR) values and receiver operating characteristic curves. The scoring model was then internally validated in validation set. The results showed that there were 11 indicators were statistically different between the two groups in the training set, including white blood cells, neutrophil-to-lymphocyte ratio, platelets, eosinophils (EO), high sensitivity C-reactive protein (hsCRP), activated partial thromboplastin time (APTT), sodium, potassium (K), albumin (ALB), Total bilirubin, and Immunoglobulin E (IgE) in the univariate analysis. Among them, the independent risk factors for GI bleeding included the six indicators of EO ≤ 0.045×10^9/L, hsCRP ≥ 14.5 mg/L, APTT ≤ 28.1 s, K ≥ 4.18 mmol/L, ALB ≤ 40.6 g/L, and IgE ≥ 136 ng/mL. According to the OR values, where EO ≤ 0.045 ×10^9/L, hsCRP ≥ 14.5 mg/L, APTT ≤ 28.1 s, ALB ≤ 40.6 g/L each scored 3 points, K ≥ 4.18 mmol/L, IgE ≥ 136 ng/mL each scored 2 points, and the total score was 0-16 points. The sensitivity and specificity of predicting GI bleeding were 88.7% and 64.2%, respectively, when the child scored ≥ 7 points. In the validation set, the sensitivity, specificity and accuracy of the model in predicting GI bleeding were 77.4%, 74.5% and 75.2%, respectively. In conclusion, the construction of a scoring model to predict the risk of GI bleeding from abdominal HSP would greatly assist pediatricians in predicting and identifying children at high risk for GI bleeding at an early stage.

## Introduction

Henoch-Schönlein purpura (HSP), also known as immunoglobulin A vasculitis (IgAV), is the most common form of vasculitis in children (1–3). HSP occurs in the fall, winter and spring, with annual incidence rates of 10/100,000 to 20/100,000 reported for those under 17 years of age (4, 5). HSP may occur in many systems of the body and can be clinically classified as abdominal, arthritic, renal and mixed types (6–8), among them, abdominal HSP accounted for 58%-77% (9, 10). Gastrointestinal (GI) symptoms of abdominal HSP were often manifested as abdominal pain, GI bleeding, nausea, and vomiting, and the appearance of GI symptoms was not synchronized with skin symptoms (11). Although GI bleeding accounted for approximately 18-52% of abdominal HSP (12, 13), GI bleeding posed a serious threat to patients’ lives (14, 15), and severe GI injuries, such as massive intestinal bleeding, were associated with significantly increased mortality (16, 17). Therefore, early recognition of GI bleeding is crucial for subsequent treatment and prognosis. GI bleeding might occur at different times in the course of the disease, and because of its insidious onset, some children presented early with only positive stool occult blood (SOB) without clinical manifestations (11, 18), making early recognition of GI bleeding in some children difficult and challenging. Therefore, it is necessary and practical to establish a predictive model for HSP GI bleeding based on objective indicators rather than relying exclusively on physicians’ personal experience, especially for junior physicians and primary care hospitals.

Several studies have been reported, such as platelet count (19), mean platelet volume (20), platelet-to-lymphocyte ratio (21), and neutrophil-to-lymphocyte ratio (NLR) (21, 22) associated with GI bleeding, and these studies provide a basis and great help for physicians to determine GI bleeding. However, the current study had certain shortcomings, mainly that it presented only a correlation between a single risk factor and GI bleeding, but due to the complexity and variability of patient examination findings, these single factors in the above study were often vulnerable to other factors, and GI bleeding itself is influenced by many factors, it is not yet possible to accurately assess the risk of GI bleeding with individual factors. There is no clear study related to the prediction model of GI bleeding in abdominal HSP, and the aim of this study was to construct a prediction model to determine whether it is GI bleeding, and to perform internal validation to test the reliability of the prediction model and improve its external generalization and use.

## Methods

**Figure 1:**
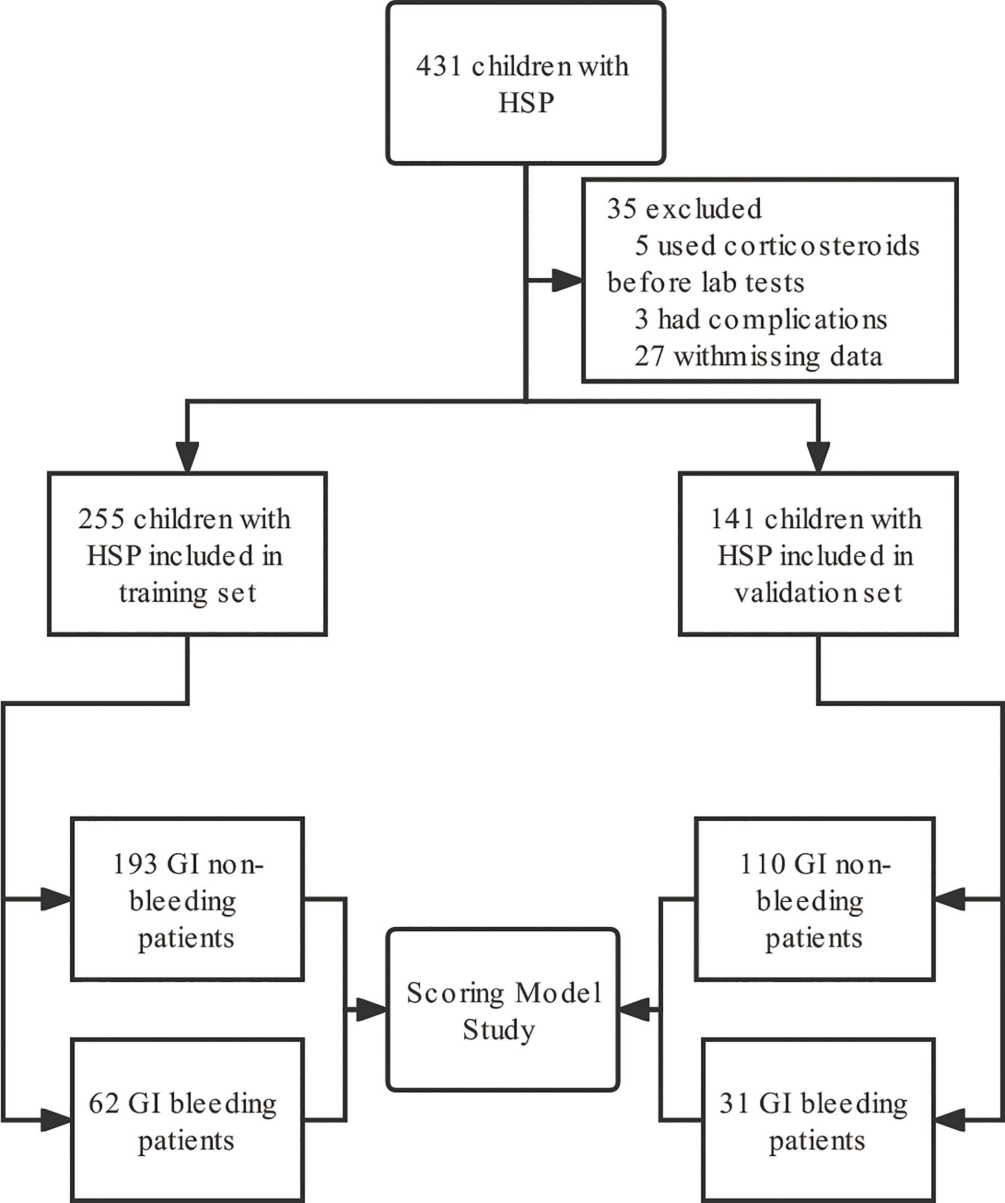
Flow chart of study subject inclusion. GI, gastrointestinal; HSP, Henoch-Schönlein purpura.

Subjects: We screened 431 children with abdominal HSP hospitalized in Wuhan Children’s Hospital from June 2020 to June 2022 for a single-center retrospective study, of whom 396 (91.8%) were eligible (**Figure 1**). The patients are divided into two parts according to the admission time, the training set and the validation set, where the total number of patients in the validation set was about 30% of the total number of patients. The training set consisted of 193 children (120 males and 73 females) with GI non-bleeding with a median age of 6.0 (5.0, 8.0) years and 62 children (42 males and 20 females) with GI bleeding with a median age of 6.0 (4.0, 8.0) years from June 2020 to October 2021. The internal validation set included children hospitalized from November 2021 to June 2022 and included 110 children with GI non-bleeding (70 males and 40 females) with a median age of 7.0 (5.0, 8.0) years and 31 children with GI bleeding (22 males and 9 females) with a median age of 5.0 (4.0, 7.0) years.

Diagnostic criteria for HSP: According to the European League Against Rheumatism, Pediatric Rheumatology International Trials Organization, and the Pediatric Rheumatology European Society, the diagnostic criteria for HSP should be the presence of at least one of the following four criteria: acute onset of diffuse abdominal pain, arthritis or arthralgia, renal involvement (hematuria and/or proteinuria), and biopsy showing major immunoglobulin A (IgA) deposition (23). Almost all patients with HSP have purpura or petechial rash with a distribution related to pressure or gravity (24, 25). In clinical work, HSP was diagnosed in children presenting with non-thrombocytopenic typical purpura and rash with multisystem (GI, renal, joint) involvement after excluding other diseases.

GI manifestations of abdominal HSP: (1) diffuse abdominal pain, including colic; (2) nausea and vomiting; (3) GI bleeding: vomiting blood, black stools, blood in the stool or positive stool occult blood; (4) bleeding gastric and duodenal ulcers confirmed by gastroscopy, and (5) rare intussusception, protein-losing enteropathy, intestinal perforation, hemorrhagic ascites, acute stone free cholecystitis and acute pancreatitis.

Inclusion criteria: HSP diagnostic criteria were met; abdominal HSP with GI manifestations; children were treated according to norms; all blood samples were collected prior to before treatment with steroids.

Exclusion criteria: Children diagnosed with sepsis, obesity, hyperlipidemia, diabetes mellitus, hypertension, chronic kidney disease, nephrotic syndrome, inflammatory bowel disease, and chronic inflammatory diseases were not included in the study, and children who had received corticosteroids prior to before blood count analysis were excluded from the study. Children with missing or incomplete medical records will also be excluded

Treatment protocols: The basic treatment strategy for HSP on admission was to recommend bed rest and symptomatic management, and to administer prednisolone 1-2 mg/kg/d orally or intravenously when the patient complained of unacceptable abdominal or joint pain.

Laboratory tests: Before the first admission, 2.0 mL of venous blood was collected in an EDTA-K2 anticoagulated vacuum blood collection tube, mixed upside down, and sent to the laboratory for testing. The XN-3000 fully automated hematology analyzer (Sysmex, Kobe, Japan) was used for the determination of routine blood flow. At the same time, a red (no procoagulant or anticoagulant added) separator vacuum tube and blue sodium citrate (9:1) anticoagulant vacuum tube were used to collect 2.0 mL of venous blood each and sent to the laboratory. Centrifugation (3300g, 5min) in a BA-600 centrifuge (Baiyang, Beijing, China) to separate serum. The serum in red blood collection tubes was tested for the biochemical parameters albumin (ALB), total bilirubin (TB), sodium (Na), and potassium (K) using a Cobas-8000 automated biochemical analyzer (Roche, Mannheim, Germany) and for high sensitivity C-reactive protein (hsCRP), Immunoglobulin E (IgE) and IgA using a BNII auto-analyzer system (Siemens, Erlangen, Germany). Measurement of prothrombin time (PT) and activated partial thromboplastin time (APTT) was performed on blue anticoagulated tube serum using a CS-5100 fully automated coagulation analyzer (Sysmex, Kobe, Japan). All specimens are tested on the machine within 2h in the laboratory department, and the test results are uploaded to the electronic medical record system after verification by a dedicated person in the laboratory department. The instruments in the laboratory are regularly calibrated and accuracy tested with coefficients of variation within normal limits, and the instruments are quality controlled daily using different concentrations of samples.

Data Collection: The study subjects were obtained from the inpatient electronic medical record system (Donghua, Beijing, China), and all children’s demographic data, including hospitalization date, hospitalization number, gender, age, height, weight, and body mass index (BMI), were recorded in the medical records. Laboratory tests included routine blood, biochemical indicators (liver function, electrolytes), infection indicators (hsCRP), coagulation function (PT, APTT), and immune function indicators IgE and IgA, which were completed on the day of admission. The above medical record information was recorded by a dedicated person and carefully proofread by another professional. The study was approved by the Ethics Committee of Wuhan Children’s Hospital, and a waiver of informed consent was granted (2022R077).

Statistical methods: Statistical analysis was performed by SPSS 23.0 (IBM, New York, USA). The Shapiro-Wilk test was used to test whether two groups of continuous variables conformed to normal distribution. For variables that were subject to the normal distribution in both groups, the difference between groups was expressed by a *t*-test with *x* ± *s*. The difference between groups of non-normally distributed variables was expressed by the Mann-Whitney *U* test with median (25^th^, 75^th^), and dichotomous variables were expressed by a four-grid table and composition ratio using the chi-square test. Variables with significant differences (*P* < 0.05) between two comparisons in univariate analysis between groups were included in multivariate analysis. Continuous variables were converted to dichotomous variables based on ease of clinical use before proceeding to the multifactorial analysis. Finally, variables with statistical differences in the univariate analysis were included in the binary logistic regression equation, independent risk factors were obtained by stepwise regression using the backward conditional method, and the goodness-of-fit was calculated for each step by the Hosmer-Lemeshow test. The odds ratio (OR) values of each independent risk factor were assigned to obtain the scoring model, the total score of each child was calculated, and the receiver operating characteristic (ROC) curve was used to calculate the best cutoff value of the total score to obtain the sensitivity and specificity of the scoring model differentiation. In the internal validation set, scores were calculated separately for each child and a four-grid table was constructed based on the cutoff values of the true clinical diagnosis and predictive scoring model to validate the sensitivity, specificity, and accuracy of the model. Statistical differences were set at *P* < 0.05.

## Results

### Demographic characteristics

We finally included 396 eligible children with HSP for analysis, with a median age of 6 (5, 8) years for whom without GI bleeding, including 190 males and 113 females, and 6 (4, 8) years for whom with GI bleeding, including 64 males and 29 females, divided into a training set and a validation set according to the time of admission. And in the training set, there was no statistical difference between the children in the HSP GI bleeding group and the GI non-bleeding group in terms of gender, age, height, weight, and BMI (*P* > 0.05, **Table 1**).

**Table 1:** Demographic characteristics of children in the GI bleeding and GI non-bleeding groups in the training set.

	GI non-bleeding	GI bleeding	Z/x^2^	*P* value
Patients (n)	193	62		
Gender (M/F)	120/73 (62.2%/37.8%)	42/20 (67.7%/32.3%)	0.627	0.428
Age (year)	6 (5 - 8)	6 (4 - 8)	-0.169	0.866
Height (cm)	122.0 (114.5 - 133.0)	123.0 (110.0 - 134.8)	-0.209	0.835
Weight (kg)	22.0 (18.0 - 29.0)	20.5 (17.5 - 28.1)	-0.655	0.512
BMI (kg/m^2^)	14.8 (13.6 - 16.8)	14.4 (13.4 - 16.0)	-1.422	0.155

GI, gastrointestinal; M/F, male/female; BMI, body mass index.

### Admission laboratory tests

#### Blood count and infection indicators

Six variables of blood routine and infection indicators were included: white blood cells (WBC), NLR, platelets (PLT), eosinophils (EO), red blood cells (RBC), and hsCRP, among which WBC, NLR, PLT, EO, and hsCRP were statistically different between the two groups (*P* < 0.05). In the GI bleeding group compared to the GI non-bleeding group, WBC (11.16 ×10^9/L *vs.* 9.83 ×10^9/L, *P* = 0.009), NLR (4.2 *vs.* 2.4, *P* < 0.001), PLT (382.0 ×10^9/L *vs.* 361.0×10^9/L, *P* = 0.016) and hsCRP (5.10 mg/L *vs.* 3.42 mg/L, *P* = 0.003) were higher, while EO (0.030 ×10^9/L *vs.* 0.090 ×10^9/L, *P* < 0.001) was lower (**Table 2**).

**Table 2:** Laboratory tests of children in the GI bleeding and GI non-bleeding groups in the training set.

	GI non-bleeding	GI bleeding	Z/x^2^	*P* value
Patients (n)	193	62		
WBC (10^9/L)	9.83 (7.45 - 13.21)	11.16 (8.74 - 15.77)	-2.623	0.009
NLR	2.4 (1.5 - 4.5)	4.2 (2.8 - 6.7)	-3.821	< 0.001
PLT (10^9/L)	361.0 (275.0 - 417.0)	382.0 (330.8 - 478.3)	-2.419	0.016
EO (10^9/L)	0.090 (0.035 - 0.180)	0.030 (0.000 - 0.103)	-4.280	< 0.001
RBC (10^12/L)	4.55 (4.29 - 4.81)	4.62 (4.28 - 4.96)	-0.510	0.610
hsCRP (mg/L)	3.42 (1.00 - 9.41)	5.10 (2.43 - 23.73)	-2.930	0.003
PT (s)	11.4 (10.7 - 12.0)	11.2 (10.8 - 11.8)	-1.005	0.315
APTT (s)	28.1 (25.3 - 30.9)	25.6 (22.9 - 28.0)	-3.645	< 0.001
Na (mmol/L)	138.4 (136.4 - 140.6)	137.6 (135.9 - 139.2)	-2.134	0.033
K (mmol/L)	4.03 (3.77 - 4.47)	4.29 (3.99 - 4.60)	-2.569	0.010
ALB (g/L)	43.0 (39.9 - 44.9)	39.8 (37.1 - 43.2)	-4.366	< 0.001
TB (μmol/L)	6.9 (4.8 - 8.7)	7.5 (5.8 - 11.0)	-2.308	0.021
IgE (ng/mL)	90.4 (33.7 - 277.0)	182.0 (58.9 - 330.1)	-2.190	0.028
IgA (g/L)	1.97 (1.49 - 2.51)	1.89 (1.44 - 2.54)	-0.579	0.563

GI, gastrointestinal; WBC, white blood cells; NLR, neutrophil-to-lymphocyte ratio; PLT, platelets; EO, eosinophils; RBC, red blood cells; hsCRP, high sensitivity C-reactive protein; PT, prothrombin time; s, seconds; APTT, activated partial thromboplastin time; Na, sodium; K, potassium; ALB, albumin; TB, total bilirubin; IgE, immunoglobulin E; IgA, immunoglobulin A.

#### Biochemical indicators

Biochemical indicators were mainly included in coagulation (PT, APTT), electrolytes (Na, K), liver function (ALB, TB), and immunoglobulin (IgE, IgA) indicators, among which the six indicators with statistical differences were APTT, Na, K, ALB, TB, IgE (*P* < 0.05). The GI bleeding group had lower APTT (25.6 s *vs.* 28.1 s, *P* < 0.001), Na (137.6 mmol/L *vs.* 138.4 mmol/L, *P* = 0.033), and ALB (39.8 g/L *vs.* 43.0 g/L, *P* < 0.001) than the GI non-bleeding group, while K (4. 29 mmol/L *vs.* 4.03 mmol/L, *P* = 0.010), TB (7.5 μmol/L *vs.* 6.9 μmol/L, *P* = 0.021), and IgE (182.0 ng/mL *vs.* 90.4 ng/mL, *P* = 0.028) were higher (**Table 2**).

### Conversion of continuous variables to dichotomous variables

A total of 11 continuous variables (WBC, NLR, PLT, EO, hsCRP, APTT, Na, K, ALB, TB, IgE) in the univariate analysis showed statistically significant differences between the GI bleeding and GI non-bleeding groups (*P* < 0.05). For clinical convenience, ROC curves were applied to calculate the cutoff values, sensitivity, and specificity corresponding to the area under curve (AUC), *P*-value and maximum Youden index for each continuous variable, while converting them into dichotomous variables according to the cutoff values, as shown in **Table 3**.

**Table 3:** The cutoff value of converting continuous variables to dichotomous variables in training set.

Cutoff value	AUC (95% CI)	*P* value	Sensitivity	Specificity
WBC ≥ 13.51×10^9/L	0.611 (0.531 - 0.690)	0.009	0.435	0.777
NLR ≥ 2.81	0.661 (0.583 - 0.739)	< 0.001	0.758	0.585
PLT ≥ 314×10^9/L	0.602 (0.526 - 0.678)	0.016	0.839	0.378
EO ≤ 0.045×10^9/L	0.680 (0.601 - 0.760)	< 0.001	0.597	0.715
hsCRP ≥ 14.5 mg/L	0.624 (0.541 - 0.706)	0.003	0.419	0.839
APTT ≤ 28.1 s	0.654 (0.577 - 0.731)	< 0.001	0.790	0.497
Na ≤ 139.5 mmol/L	0.590 (0.513 - 0.667)	0.033	0.823	0.363
K ≥ 4.18 mmol/L	0.608 (0.528 - 0.689)	0.010	0.645	0.611
ALB ≤ 40.6 g/L	0.684 (0.609 - 0.760)	< 0.001	0.645	0.705
TB ≥ 9.1 μmol/L	0.597 (0.516 - 0.679)	0.021	0.403	0.793
IgE ≥ 136 ng/mL	0.592 (0.510 - 0.675)	0.029	0.597	0.580

AUC, area under curve; CI, Confidence Interval; WBC, white blood cells; NLR, neutrophil-to-lymphocyte ratio; PLT, platelets; EO, eosinophils; hsCRP, high sensitivity C-reactive protein; APTT, activated partial thromboplastin time; s, seconds; Na, sodium; K, potassium; ALB, albumin; TB, total bilirubin; IgE, immunoglobulin E.

### Independent risk factors and construction of scoring models

**Figure 2:**
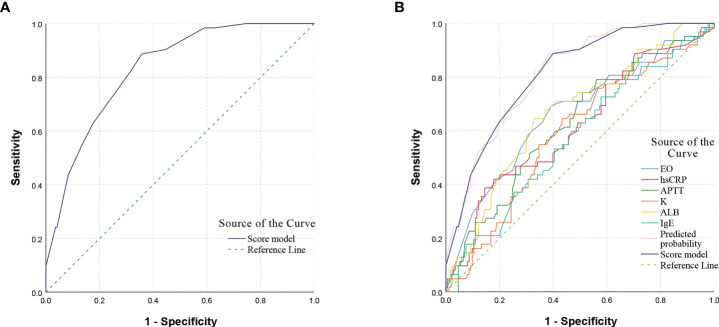
ROC curve of scoring model and single indicator. **(A)** ROC curve of the scoring model. The AUC (95% CI) for predicting GI bleeding was 0.832 (0.779, 0.884), *P* < 0.001. **(B)** ROC curve of the single indicator. A comparison of the AUC of the ROC curves for different indicators showed that the AUC of the scoring model was higher than that of the single indicator. The AUC (95% CI) for predicted probability was 0.833 (0.781, 0.885), *P* < 0.001, and the difference in AUC values between the scoring model and the predicted probability was not significant. ROC, receiver operating characteristic; AUC, area under the curve; CI, confidence interval.

The previous 11 variables were included in the binary logistic regression equation and the backward conditional stepwise regression finally yielded 6 variables (EO, hsCRP, APTT, K, ALB, IgE) as their independent influencing factors, where the OR (95% Confidence Interval, CI) value for the diagnosis of GI bleeding with EO ≤ 0.045×10^9/L was 3.400 (1.714 - 6.748), *P* < 0.001; OR (95% CI) for the diagnosis of GI bleeding with hsCRP ≥ 14.5 mg/L was 2.837 (1.334 - 6.035), *P* = 0.007; OR (95% CI) for the diagnosis of GI bleeding with APTT ≤ 28.1 s was 3.426 (1.563 - 7.510), *P* = 0.002; OR (95% CI) for K ≥ 4.18 mmol/L for the diagnosis of GI bleeding was 2.288 (1.154 - 4.535), *P* = 0.018; OR (95% CI) for ALB ≤ 40.6 g/L for the diagnosis of GI bleeding was 3.476 (1.739 - 6.946), *P* < 0.001; and the OR (95% CI) for the diagnosis of GI bleeding with IgE ≥ 136 ng/mL was 2.246 (1.135 - 4.443), *P* = 0.020. For ease of clinical use, these values are assigned as approximate proportions. We define 3 points for EO ≤ 0.045×10^9/L and 0 points for EO > 0.045×10^9/L; 3 points for hsCRP ≥ 14.5 mg/L and 0 points for hsCRP < 14.5 mg/L; 3 points for APTT ≤ 28.1 s and 0 points for APTT > 28.1 s; 2 points for K ≥ 4.18 mmol/L and 0 points for K < 4.18 mmol/L; 3 points for ALB ≤ 40.6 g/L, 0 points for ALB > 40.6 g/L; 2 points for IgE ≥ 136 ng/mL, 0 points for IgE < 136 ng/mL (**Table 4**). The score prediction model was constructed with the above six indicators, and each patient could score a total of 0 - 16 points. The total score was calculated for each child, and then the ROC curve was used to calculate the maximum Youden index for the total score corresponding to a cutoff value of 7, with an AUC (95% CI) of 0.832 (0.779, 0.884), *P* < 0.001. When the score of the scoring prediction model was ≥ 7 points, the sensitivity and specificity of predicting GI bleeding were 88.7% and 64.2%, respectively (**Figure 2**).

**Table 4:** OR values and scores of binary logistic regressions in the training set.

Variable(s)	*P* value	OR values (95% CI)	Point
EO ≤ 0.045×10^9/L	< 0.001	3.400 (1.714 - 6.748)	3
hsCRP ≥ 14.5 mg/L	0.007	2.837 (1.334 - 6.035)	3
APTT ≤ 28.1 s	0.002	3.426 (1.563 - 7.510)	3
K ≥ 4.18 mmol/L	0.018	2.288 (1.154 - 4.535)	2
ALB ≤ 40.6 g/L	< 0.001	3.476 (1.739 - 6.946)	3
IgE ≥ 136 ng/mL	0.020	2.246 (1.135 - 4.443)	2

OR, Odds ratio; CI, Confidence Interval; EO, eosinophils; hsCRP, high sensitivity C-reactive protein; APTT, activated partial thromboplastin time; s, seconds; K, potassium; ALB, albumin; IgE, immunoglobulin E.

### Internal validation

When the score was ≥ 7 points, the sensitivity of predicting GI bleeding was 77.4%, the specificity was 74.5%, and the accuracy was 75.2% (**Table 5**).

**Table 5:** Internal validation of scoring model.

Score (point)	GI non-bleeding	GI bleeding	Total
≥ 7 points	28	24	52
< 7 points	82	7	89
Total	110	31	141

GI, gastrointestinal.

## Discussion

In this study, we constructed and internally validated a scoring model for early prediction of the risk of abdominal HSP gastrointestinal bleeding by analyzing children with abdominal HSP hospitalized in Wuhan Children’s Hospital. The model consisted of six variables, EO, hsCRP, APTT, K, ALB and IgE, with EO ≤ 0.045 ×10^9/L, hsCRP ≥ 14.5 mg/L, APTT ≤ 28.1 s and ALB ≤ 40.6 g/L each scoring 3 points, and K ≥ 4.18 mmol/L and IgE ≥ 136 ng/mL each scoring 2 points, for a total score of 0 to 16. When the score was ≥ 7 points, the sensitivity and specificity of predicting GI bleeding were 88.7% and 64.2%, respectively. The sensitivity, specificity, and accuracy of the model in predicting GI bleeding were 77.4%, 74.5%, and 75.2%, respectively, when validated internally by children admitted at different times, suggesting that the model has some value for use and promotion.

There have been many reports on the relationship between organ involvement and laboratory indicators in HSP patients (26, 27), but they have all investigated whether some scattered laboratory indicators were risk factors for organ involvement in HSP patients, and to our knowledge no study has constructed and validated a predictive model that combined multiple indicators. One of the limitations of individual risk factors to predict outcome is that they are susceptible to confounding factors, and different centers, populations, and regions may reach opposite conclusions. Regarding laboratory risk factors and organ involvement in HSP, NLR was by far one of the most numerous and important risk factors (21, 22, 26, 27). Most findings suggested that high NLR was associated with GI and renal involvement in HSP (22, 26, 27), however, some authors have come to the opposite conclusion (21). In our results, the NLR was also higher in the GI bleeding group than in the GI non-bleeding group (4.2 vs. 2.4), and the difference was statistically significant (*P* < 0.001). After regression analysis, NLR did not enter the final prediction model. It was suggested that prediction by NLR alone may have limitations for different regions or populations. Therefore, we constructed a prediction model including several indicators, namely EO, hsCRP, APTT, K, ALB and IgE for a total of six indicators.

In our scoring prediction model, the first predictor was EO, there has been no clear report on EO and HSP GI bleeding before. In the model, EO ≤ 0.05 × 10^9/L was an independent risk factor for predicting GI bleeding and scored 3 points, which suggested that reduced EO may have a suggestive effect on the appearance of GI bleeding in abdominal HSP. hsCRP was an indicator of infection and the current results suggested a significant increase in children in the GI bleeding group (*P* = 0.003), which was consistent with previous results (28–30), so hsCRP ≥ 14.5 mg/L also scored 3 points in the current model. Previous studies had shown that coagulation in children with HSP was approximately within the normal range (31), and the APTT (25.6 s *vs.* 28.1 s, *P* < 0.001) and PT (11.2 s *vs.* 11.4 s, *P* = 0.315) in children with and without GI bleeding in the current model were within the normal range, which was also consistent with previous studies and the clinical presentation of HSP. We also found that the APTT was shorter in children with GI bleeding than in children without GI bleeding, and the difference was statistically significant. Although APTT was within the normal range, it may have some discriminatory significance for the presence of GI bleeding. In the scoring model, the final calculation yielded a score of 3 points for APTT ≤ 28.1 s. ALB was usually associated with HSP renal disease (32), and the relationship with HSP GI bleeding was not clear, but some severe GI bleeding would present with reduced ALB and even require ALB supplementation (33), suggesting that reduced ALB may be of some value in identifying the presence of GI bleeding. Therefore, we obtained a score of 3 points for ALB ≤ 40.6 g/L in the model as well. The other two indicators scored 2 points each, K ≥ 4.18 mmol/L and IgE ≥ 136 ng/mL, respectively. The significance of serum K in HSP gastrointestinal bleeding has not been reported, but the occurrence of bleeding may lead to elevated K levels as K was released from the red blood cells and reabsorbed (34). Our study showed that children in the GI bleeding group had higher blood K compared to those in the GI non-bleeding group (4. 29 mmol/L *vs.* 4.03 mmol/L, *P* = 0.010), and K ≥ 4.18 mmol/L scored 2 points in the scoring model. This may be due to K reabsorption in children with gastrointestinal bleeding, but more studies may be needed to clarify. Previous studies have confirmed that children with HSP may have elevated IgE (35), and our results also suggest that both GI bleeding and GI non-bleeding children showed elevated IgE (182.0 ng/mL *vs.* 90.4 ng/mL, *P* = 0.028), but the elevated IgE was more pronounced in GI bleeding children and IgE ≥ 136 ng/mL scored 2 points in the scoring model. In summary, a scoring model with the six early indicators mentioned above may help clinicians to assess and determine the risk of GI bleeding early, although more studies and reports are needed to confirm the exact mechanism.

Of course, there were some limitations in our study: (1) our definition of GI bleeding classification depends on the description of medical history, gastroscopy and stool examination, and some children may be missed; (2) the etiology of HSP itself was not completely clear, and the specific mechanisms of some predictive indicators were not clear, and our study only provided a clinical prediction model, and the specific mechanisms need to be studied more thoroughly; and (3) our study was based on a retrospective study of single-center hospitalized children only. There was a selection bias, and future multicenter or even prospective studies may be needed to help improve the assessment model. Nevertheless, the significance of the model in this study was that by using multiple factors to predict the risk of GI bleeding, the subjective assessment and personal experience of physicians could be reduced, especially for some primary and community hospitals where gastroscopy could not be perfected, and can provide an additional option for the possible early assessment of bleeding that is worth promoting.

## Conclusion

GI bleeding in HSP may occur on any day of the disease course and the early symptoms are atypical, but some children may bleed severely and even life-threateningly. Therefore, we constructed a scoring prediction model for abdominal HSP GI bleeding to identify and evaluate children with possible GI bleeding through some early laboratory tests to assist physicians in the next step of decision-making and treatment.

## Data availability statement

The raw data supporting the conclusions of this article will be made available by the authors, without undue reservation.

## Ethics statement

The studies involving human participants were reviewed and approved by Ethics Committee of Wuhan Children’s Hospital. Written informed consent from the participants’ legal guardian/next of kin was not required to participate in this study in accordance with the national legislation and the institutional requirements.

## Author contributions

YS conceived and designed the project and directed the revision of the paper. LS and WL collected and organized the data. CL performed the writing of the manuscript, and YZ directed the statistical methods and analyzed and interpreted the data. All authors had access to the primary data and were responsible for the accuracy and completeness of the results. Final responsibility for the decision to submit the paper. All authors were responsible for the content of this paper.

## Conflict of interest

The authors declare that the research was conducted in the absence of any commercial or financial relationships that could be construed as a potential conflict of interest.

## Publisher’s note

All claims expressed in this article are solely those of the authors and do not necessarily represent those of their affiliated organizations, or those of the publisher, the editors and the reviewers. Any product that may be evaluated in this article, or claim that may be made by its manufacturer, is not guaranteed or endorsed by the publisher.
